# Method for AFM investigation of lateral forces required for the surface detachment of neurites

**DOI:** 10.1038/s41598-026-60250-1

**Published:** 2026-07-07

**Authors:** Vinzent Braemer, Gerrit Paasche

**Affiliations:** 1 Department of Otorhinolaryngology, Head and Neck Surgery, Hannover Medical School, 30625 Hannover, Germany; 2 Lower Saxony Center for Biomedical Engineering, Implant Research and Development, 30625 Hannover, Germany; 3 Hannover Medical School, Hearing4all Cluster of Excellence, 30625 Hannover, Germany

**Keywords:** Atomic force microscopy, Lateral detachment force, Adhesion strength, Spiral ganglion neurons, Neuronal outgrowth, Mixed culture purification, Biological techniques, Cell biology, Materials science, Neuroscience

## Abstract

Resprouting of neurites to establish direct connections between stimulation electrodes and spiral ganglion neurons is of considerable interest in current cochlear implant research. The adhesion strength of neurites to implant surfaces is a critical factor determining whether such connections develop, remain stable or are disrupted shortly after formation. In this study, an in vitro protocol was established to quantify the lateral forces required to detach neurites from the surface of precoated substrates using atomic force microscopy. Spiral ganglion neurons were isolated from mixed primary cell cultures using cytosine β-D-arabinofuranoside hydrochloride as a mitotic inhibitor. After several days of cultivation, the neurites were mechanically hooked with the cantilever tip and laterally dragged across the substrate surface to measure the forces required for detachment. Four different parameters and their influence on the detachment process were evaluated: exposure time to measurement conditions, the distance between growth cone and cantilever, the distance between cantilever and soma, and the total length of the neurite. The mean lateral detachment force was 2.47 ± 0.19 nN, and no significant correlation was observed between the lateral forces and any of the four investigated parameters. Overall, this study provides quantitative information on neurite-surface adhesion behaviour and strength in vitro.

**Supplementary Information:**

The online version contains supplementary material available at 10.1038/s41598-026-60250-1.

## Introduction

Modern cochlear implant (CI) research focusses on improving signal transmission, stimulation accuracy and overall device performance^1,2^. During surgical insertion of the implant electrode array into the cochlea, friction and shear forces can cause permanent damage to the delicate biological structure^3,4^. Insertion-related trauma includes tearing of the basilar membrane, which houses inner and outer hair cells, as well as possible disruption of spiral ganglion fibers due to fractures of the osseous spiral lamina^5^. Such injuries to the inner ear can lead to degeneration of spiral ganglion neurons (SGNs) and loss of patient’s residual hearing^6^.

While some approaches aim to mitigate electrode insertion related hearing loss during implantation with immediate administration of dexamethasone supplemented artificial perilymph^7^, others focus on promoting neuronal survival and resprouting through the administration of neurotrophic factors such as brain-derived neurotrophic factor and neurotrophin-3^8^ or by modifying the implant surface with a biodegradable poly(4-hydroxybutyrate) coating^9^. Preservation or restoration of the neuronal structure within the cochlea is important for the CI performance^10^. In particular, neurite outgrowth toward the stimulation electrodes, thereby reducing the implant-tissue interface gap, may further enhance charge transmission and stimulation efficiency^11–13^.

Successful resprouting of spiral ganglion neurons (SGNs) and subsequent contact with cochlear implant electrodes make the adhesion strength of neurites a critical parameter to investigate. Because in vivo quantification of neurite adhesion is currently not feasible, alternative in vitro approaches are required to assess whether permanent attachment of neurites to implant surfaces is possible. Ideally, the electrode array is inserted into the scala tympani, which is filled with perilymph^14^. Movement of the patient’s head or vibrations transmitted through the oval window by functional ossicles can induce perilymphatic turbulence^15^. These movements may generate shear forces acting on adherent neurites, potentially disrupting the connection between the implant and SGNs. To date, studies have primarily focused on the topographical effects of applied shear forces on neurite outgrowth and the ability of neurons to adapt to such conditions^16,17^. However, quantitative measurements of neurite adhesion forces to substrates have not yet been reported.

Primary SGNs are typically accompanied by various non-neuronal cells, such as glia cells and fibroblasts, which provide neurotrophic support essential for neuronal survival and outgrowth^18^. Neurites are often ensheathed by these supporting cells, thereby making it difficult to target individual neurites without interfering with adjacent cells. To enable quantification of neurite attachment to non-biological substrates, isolation of individual neurites is therefore required. Cytosine β-D-arabinofuranoside hydrochloride (AraC) is a selective DNA synthesis inhibitor used for proliferation inhibition of fibroblasts and glial cells^19,20^. Prolonged exposure to AraC in the culture medium allows for the gradual isolation and purification of the SGN from mixed cell culture, enabling measurements on isolated neurites.

Atomic force microscopy (AFM) has been widely used to investigate mechanical properties and adhesion strengths of living cellular or bacterial structures in vitro^21–26^. Two principal measurement approaches have been established. The first, known as single-cell force spectroscopy (SCFS), involves either attaching individual cells to the cantilever, pressing them onto a substrate for a defined contact time, and subsequently retracting the cantilever, or detaching substrate adherent cells upon retraction after they attached to the cantilever^25,26^. In the second approach, controlled forces are applied directly to adherent cells using the cantilever tip during conventional topographical scanning. With this method cells can be detached from the substrate while their mechanical responses are recorded simultaneously^27,28^.

In the present study, AFM was used to apply shear forces to isolated neurites from primary SGNs in mixed cell cultures in order to quantify the lateral forces required to detach neurites or even neuronal cells from a non-biological substrate.

## Methods

### SGN purification

#### Substrate preparation

Circular coverslips (Ø 20 mm; neoLab Migge GmbH, Heidelberg, Germany) were prepared for cell cultivation by sterilization in an autoclave (Laboklav 25-M; Steriltechnik AG, Schloss Detzel, Germany) at 121 °C for 20 min. Afterwards, the coverslips were transferred into Nunclon 12-multiwell culture plates (Thermo Fisher Scientific, Darmstadt, Germany). Coating of the coverslips was administered with Poly-D/L-ornithine (0.1 mg/mL; Sigma Aldrich, Taufkirchen, Germany) and naturel mouse laminin (0.01 mg/mL; Invitrogen, Karlsruhe, Germany) to enhance cell adhesion. The experimental wells were filled with 1.5 mL of phosphate buffered saline solution (PBS; Invitrogen) and stored at 4 °C until usage.

#### SGN cultivation

Primary spiral ganglion neurons (SGNs) for cell cultivation were dissected from early postnatal Sprague Dawley rats (3–4 days old) of both sexes. The animals were kept in an in-house colony and separated from their mothers 30 min before cell preparation. All experiments involving primary cells were carried out in compliance with the German “Law on Protecting Animals” (§4) and the European Directive 2010/63/EU governing the use of animals for scientific purposes. All procedures received approval from the local authorities (Lower Saxony State Office for Consumer Protection and Food Safety [LAVES], Oldenburg, Germany) and were registered under number 2023/251.

The rats were rapidly decapitated using surgical scissors and the cranial cavity further processed following the protocol described by Schwieger et al.^6^. Briefly, under a reflected light microscope (MZ6; Leica Biosystems, Wetzlar, Germany) the skin was removed from the skull, and the skull was divided into two halves to allow extraction of the cochleae. The SGNs were then separated from the stria vascularis and the organ of Corti and transferred into ice-cold Panserin 401 (Pan Biotech, Passau, Germany).

Next, the enzymatic dissociation of the SGN was initiated by replacing the cooled Panserin 401 with pre-warmed Panserin 401 supplemented with 0.1% trypsin (Biochrom, Berlin, Germany) and 0.01% DNase I (Roche, Mannheim, Germany), followed by incubation for 15 min at 37 °C and 5% CO_2_. Intermittent shaking facilitated the dissociation process. After incubation, most of the digestion solution was aspirated, and 200 µL of pre-warmed fetal calf serum (FCS; Bio&Sell, Berlin, Germany) was added to terminate the enzymatic reaction. The cell cluster was then washed three times with 1 mL of supplemented Panserin 401 containing insulin (8.7 µg/mL; Biochrom), penicillin (30 U/mL; Biochrom), glucose (0.15%; B. Braun, Melsungen, Germany), PBS (0.172 mg/mL), HEPES buffer (23.43 µM; Invitrogen), and N2 supplement (0.1 µL/mL; Invitrogen).

To achieve complete dissociation of the SGN, mechanical resuspension in 1 mL of supplemented Panserin 401 using two different pipette sizes (200 and 1000 µL) was performed until no visible cell clusters remained. The cell number was determined by exclusion test with trypan blue and a Neubauer chamber. In the next step, the PBS in the pre-warmed 12-multiwell plate with the coated coverslips was aspirated and 50 × 10^4^ cells/well were seeded into each experimental well. The SGN were incubated at 37 °C, 5% CO_2_ and 95% humidity for 9–14 days in 1.5 mL (each well) of Panserin 401 supplemented with 10% FCS and 1.4 µg/mL (5 µM) AraC (Sigma Aldrich). Medium was exchanged and images of the purification development using an inverted microscope (CKX41; Olympus, Hamburg, Germany) were taken every second day.

Wells to verify neuronal survival and neurite outgrowth upon exposure to the replication inhibitor were fixated and stained after 9 days of incubation. Fixation was performed using a 1:1 acetone/methanol solution and staining using immunocytochemical labelling as described by Wefstaedt et al.^29^. Summarized concisely, a monoclonal mouse anti–200 kDa neurofilament antibody (Novocastra™, Leica) served as the primary antibody and detection was performed with the Vectastain® Elite® ABC Kit (Vector Laboratories, California, USA), followed by visualization using a DAB peroxidase substrate kit (Vector Laboratories).

### Neurite ablation

After 9 days of cultivation, the coverslips with the adherent SGN were removed from the 12-multiwell plate using fine forceps and transferred into a Petri dish (60 × 15 mm; BD Falcon, Sparks, USA) filled with PBS. The neurites were analyzed without CO_2_ regulation and at room temperature using an AFM system (NanoWizard 2, Bruker Nano GmbH, Berlin, Germany) with the same cantilever used for all detachment experiments. Prior to each measurement, the cantilever was individually calibrated to minimize external influences from different measuring time points, such as changes in ambient temperature, on the reported forces. Calibration was performed using the JPK Scanning Probe Microscope control program (Bruker Nano GmbH). For this purpose, a cleaned glass cover slip was placed beneath the cantilever. The laser spot used for detecting the cantilever deflection was centered on the cantilever, and the reflection mirror adjusted until the photodiode signal reached its maximum. Subsequently, the laser spot was vertically and horizontally aligned to the center of the photodiode. Using the force spectroscopy mode, the cantilever was brought into contact with and retracted from the glass substrate surface, while the vertical deflection as a function of measured height was recorded. These curves were used by calibration manager to determine the sensitivity of the cantilever. In the last calibration step, the spring constant was calculated by the program based on the sensitivity and the pre-measured resonance frequency of the cantilever.

For the detachment analysis, the Petri dish with the cells was positioned under the AFM head, and the cantilever was prewetted with a droplet of PBS to prevent air entrapment. Measurements were performed, using the middle cantilever of a three-cantilever system (HQ:CSC37/NoAl; NanoAndMore GmbH, Wetzlar, Germany) with a nominal spring constant of 0.3 N/m and resonance frequency of 20 kHz. Neurites were detected under an inverted light microscope (Oberserver D1, Zeiss, Oberkochen, Germany). The movement and scanning parameters were determined in the contact manipulation mode of the JPK Scanning Probe Microscope control programm (Bruker Nano GmbH). The cantilever tip was pressed onto the coverslip with an initial normal force of 3 nN and dragged over the end of a neurite with a scan speed of 20 µm/s for a maximal distance of 100 µm. If hooking of the neurite failed the normal force was increased gradually to 5 and 10 nN. Additionally, if movement or deformation of the neurite was observed but hooking was unsuccessful, a different neurite was selected to avoid potential influence from partially detached neurites. Simultaneously to the scanning process, the vertical and lateral displacement and total height of the cantilever tip was measured by a real time oscilloscope with a frequency of 25 Hz. The detachment process was documented by a LCD camera (DFK 31AF03; The Imaging Source Europe GmbH, Bremen, Germany) attached to the microscope. The whole system was positioned on an active vibration isolation platform (Accurion Halcyonics i4; Park Systems GmbH, Göttingen, Germany) to prevent external vibrations from affecting the measurements. For analysis, only neurites, which were completely detached from the coated surface, were included. In total, neurites from five cover slips on four different days were analyzed in sessions lasting 1.5 h to 4 h. After every measurement session, the cantilever was carefully submerged in 70% ethanol to remove biological residues and subsequently rinsed with deionized water to prevent salt crystal formation from residual PBS solution during drying.

### Determination of adhesion force

With the aim of calculating the shear forces required for ablation of cells from the surface, Deupree et al.^22^ presented a method, which was later modified by Zhang et al.^24^. Equation (1) describes the determination of the lateral detachment force as a function of the total compression of the cantilever, the probe geometry and the cantilever orientation:

1 $$F_{{{\mathrm{lat}}}} = F_{{{\mathrm{total}}}} \sin \left( {\theta + \Phi } \right)\cos \left( {\theta^{\prime}} \right)$$

${\mathrm{F}}_{\mathrm{l}\mathrm{a}\mathrm{t}}$ is the lateral detachment force, ${\mathrm{F}}_{\mathrm{t}\mathrm{o}\mathrm{t}\mathrm{a}\mathrm{l}}$ is the total compression of the cantilever at the time of detachment, $\uptheta$ and $\Phi$ describe the probe geometry and cantilever orientation angles (both 10° for the cantilever used) and ${\uptheta }^{^{\prime}}$ is the angle between the applied force and the lateral detachment force.

**Fig. 1:**
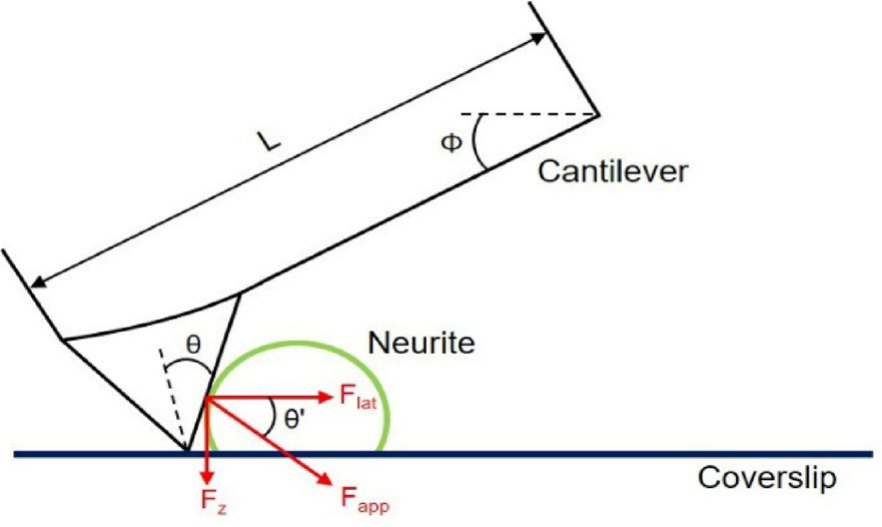
Schematic illustration of the interaction from AFM cantilever applying a shear force onto the neurite.

${\uptheta }^{^{\prime}}$ can be calculated as follows:

2 $$\theta^{\prime} = \Phi + \theta - 2\;\arctan \frac{{L - \sqrt {H_{{{\mathrm{total}}}}^{2} + \left( {L\cos \Phi } \right)^{2} } }}{{H_{{{\mathrm{Total}}}} + L\sin \Phi }}$$

where $\mathrm{L}$ is the length of the cantilever and ${\mathrm{H}}_{\mathrm{T}\mathrm{o}\mathrm{t}\mathrm{a}\mathrm{l}}$ is the measured height at the time of detachment (Fig. 1).

### Time dependency

During AFM measurements, the coverslip containing the cells was immersed in PBS at room temperature under non-sterile conditions. As these conditions may have affected cell viability and, consequently, cell adhesion to the substrate, the time (in seconds) from the initial overall measurement to the time point of detachment for each individual neurite was recorded and compared with the lateral force required for detachment.

### Distance dependency

**Fig. 2:**
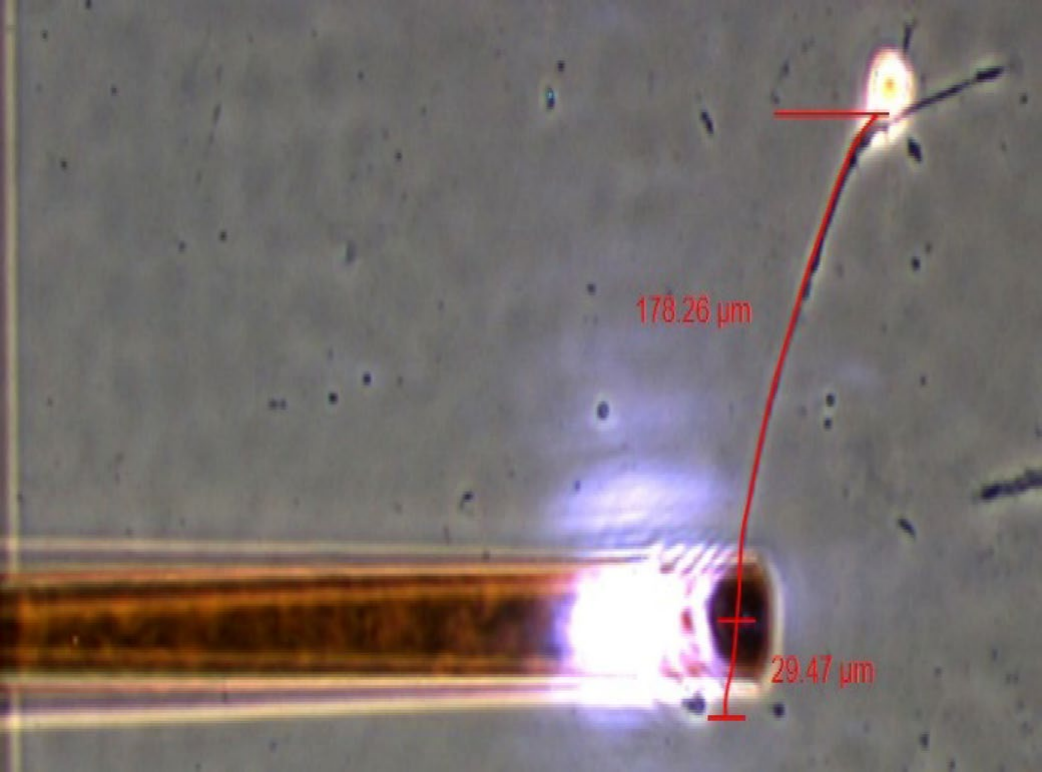
Measuring process of the distances in the detachment process. The cantilever tip was defined to be located at the midpoint of the cantilever width, and the distances from this point to the growth cone of the neurite and to the neuronal soma was measured. The sum of these distances represents the total neurite length.

Although all neurites were detached near their growth cone, the distance between the growth cone and the cantilever tip was not identical across them. Furthermore, each neurite differed in length resulting in different distances between the cantilever tip and the neuronal soma. To assess whether any of these three distances influenced the lateral force required to detach the neurite from the substrate, each distance was measured individually using the polygon function of an image analysis program (cellSens, Olympus) (Fig. 2) and compared.

### Statistics

Statistical analysis was performed using GraphPad Prism version 9.0.0 (GraphPad Software, Boston, USA). In the following results, data are presented as mean ± standard error of mean (SEM). Simple linear regression and a t-test for the null hypothesis (slope is significantly different from zero) were performed. *p*-values below 0.05 were considered statistically significant.

## Results

The following results present the purification process of the SGN culture using the mitotic inhibitor AraC and more clearly define the cellular structures targeted for the detachment measurements. Lateral detachment values were calculated using the equation described above and are presented as mean ± SEM.

### SGN purification

For the documentation of the purification progress, images of a representative region in a control well were acquired every two days, prior to medium exchange, over eight consecutive days (Supplementary Fig. 1). The images show a systematic detachment of supporting cells from the substrates surface. Changes in cell culture were most pronounced during the first 2–4 days, with fewer cells remaining at later time points. Overall, purification of the SGN culture was successfully achieved by the addition of AraC to the cell culture medium.

**Fig. 3:**
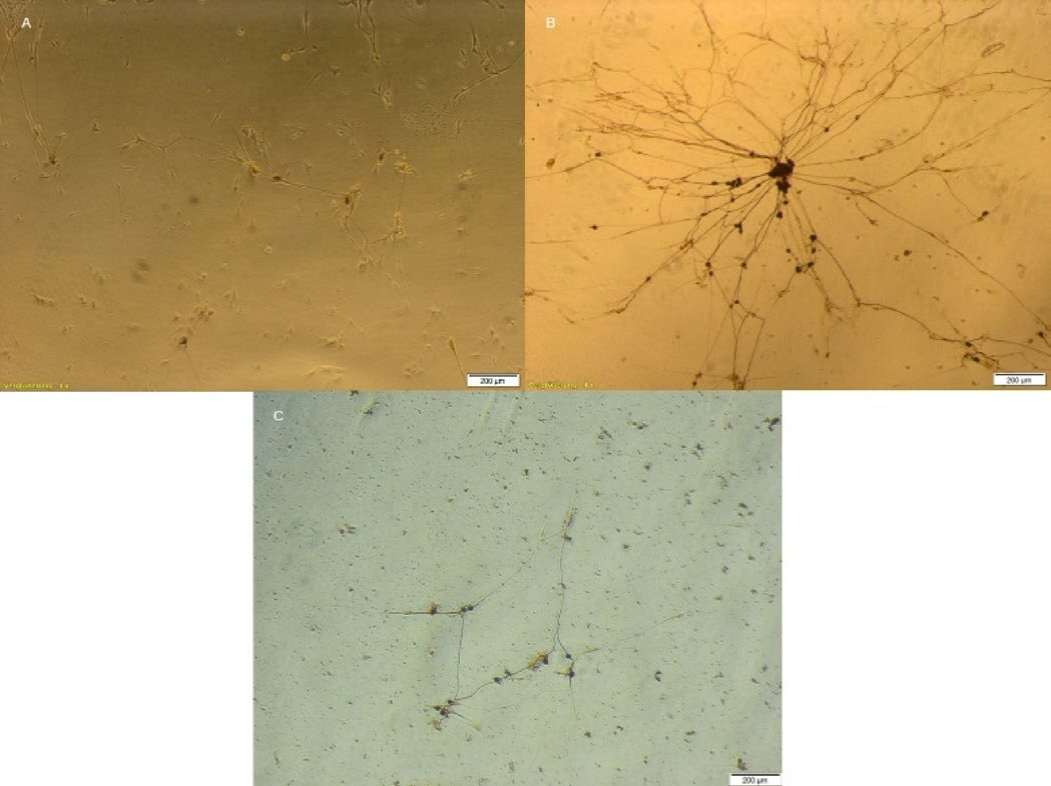
Illustrations of different regions of interest after 9 days of cultivation with AraC. (**A**) Image representing one region after SGN purification process and DAB staining. Neurons and their neurites appear brown. (**B**) Illustration of a neuron cluster with large neurite outgrowth after 9 days of cultivation and DAB staining. (**C**) Image of neurons with neurite outgrowth targeted for the detachment analysis.

The representative well was fixated after nine days of cultivation and subjected to DAB staining (Fig. 3A). As purification progressed, the staining confirmed that residual neurons survived the nine-day cultivation period and exhibited neuronal outgrowth. Moreover, only a small number of residual supporting cells are detectable after the cultivation period. Additional control wells were stained and analyzed after nine days to further verify the purification process with respect to neuronal survival and neurite outgrowth. One example of a large neuronal cluster observed is illustrated in Fig. 3B. The image displays pronounced neurite outgrowth with almost no residual non-neuronal cells remaining. Although these findings provide evidence of successful purification, such clusters were not suitable for detachment measurements. In these regions, neurites were interconnected or overlapping, resulting in reduced accessibility for the cantilever and fewer opportunities for detaching isolated neurites. Targeted neurons for the detachment measurements were less clustered ideally isolated and typically exhibited one to two neurites that were accessible and free of cells or neurites in the surrounding area (Fig. 3C). These neurites could be readily identified without staining and were therefore suitable candidates for the detachment procedure.

To assess whether a longer cultivation period would further improve the purification process, another experimental well was cultivated 14 days in AraC-supplemented Panserin 401 (Supplementary Fig. 2). While cellular networks were still detectable after 5 days of cultivation, almost no surviving cells or neuronal outgrowth were observed by day 14.

### Adhesion force

**Fig. 4:**
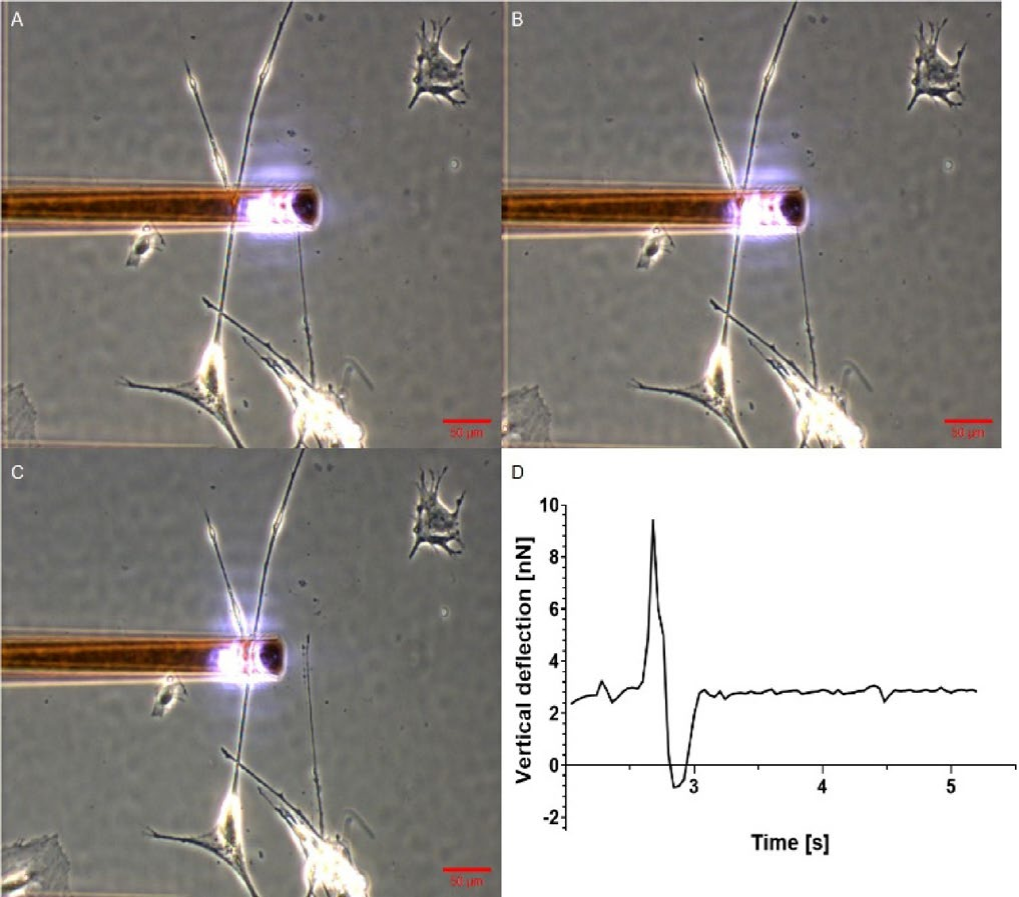
Example of a neurite detachment attempt where the initial normal force (3 nN) was too low to hook and detach the neurite. (**A**) Starting point of the detachment approach; (**B**) intermediate frame illustrating the neurite bending; (**C**) end point of the detachment attempt with the neurite end still attached to the substrate and (**D**) real time oscilloscope graph recording the vertical deflection over the time for the whole detachment attempt.

Determining the normal force required for hooking the neurites with the cantilever tip to enable a detachment was one of the main objectives in this study. When the applied force was too low, the cantilever passed over the neurite without detaching it from the surface (Fig. 4A–C). In the vertical deflection graph (Fig. 4D), an increase in force is observed at 2.6 s, indicating initial contact between the cantilever and the neurite. The force reached a maximum at 2.68 s, followed by a sharp decrease. This drop indicated either successful detachment of the neurite or slippage of the cantilever tip over the neurite. However, as the image acquired at the end of the cantilever scan (Fig. 4C) showed that the neurite was not successfully detached, the sharp force decrease was attributed to the cantilever tip losing contact with the neurite and passing over it.

**Fig. 5:**
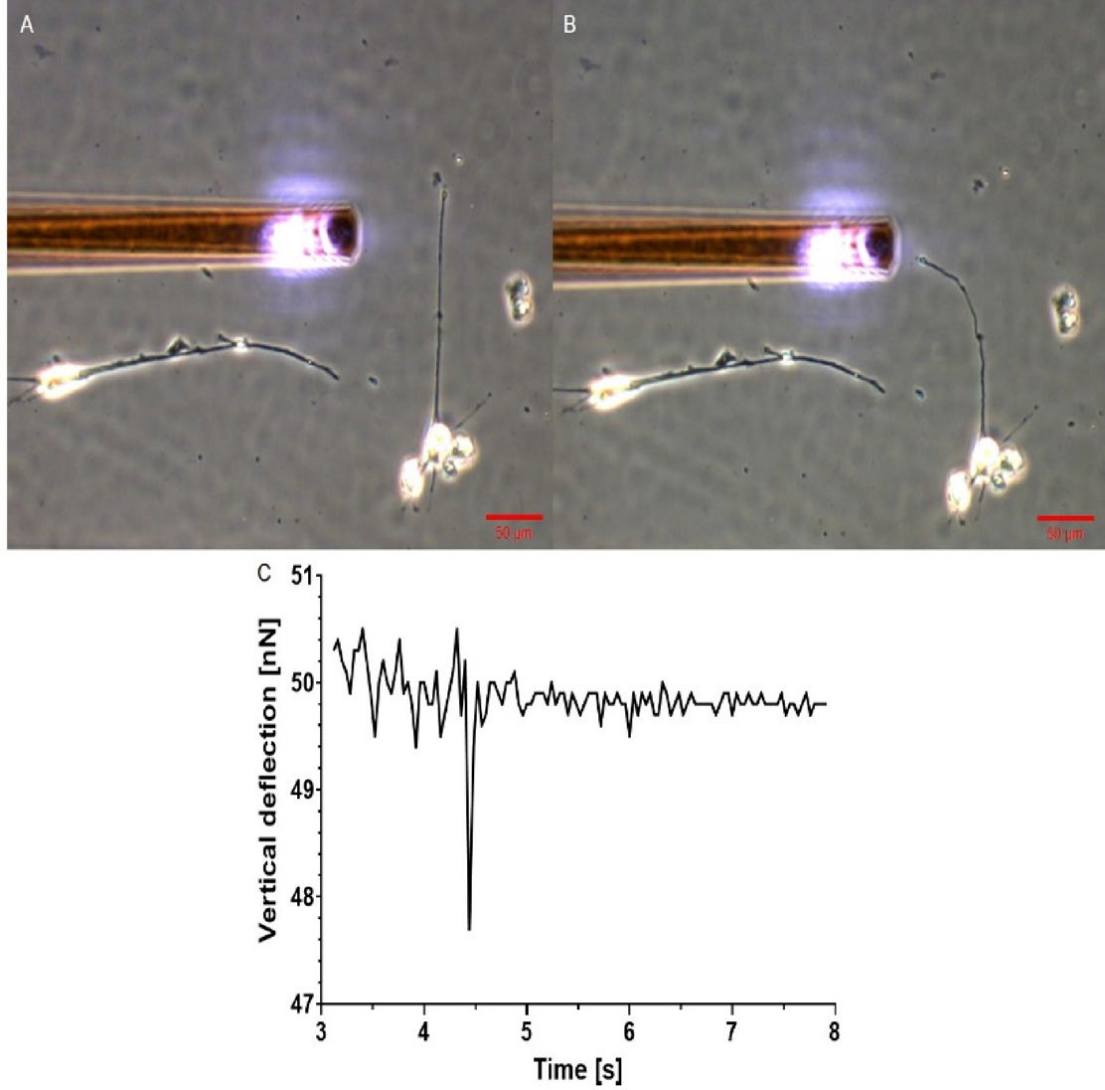
Example of a neurite detachment attempt where the initial normal force (50 nN) was too high to determine the exact moment of detachment. (**A**) Neurite and cantilever before the detachment attempt; (**B**) end point of the detachment attempt with the neurite detached and (**C**) real time oscilloscope recordings for the vertical deflection over time.

On the other hand, if the force was too high, the cantilever detached the neurite (Fig. 5A, B) but no clear peak was recorded in the real time oscilloscope force (Fig. 5C).

**Fig. 6:**
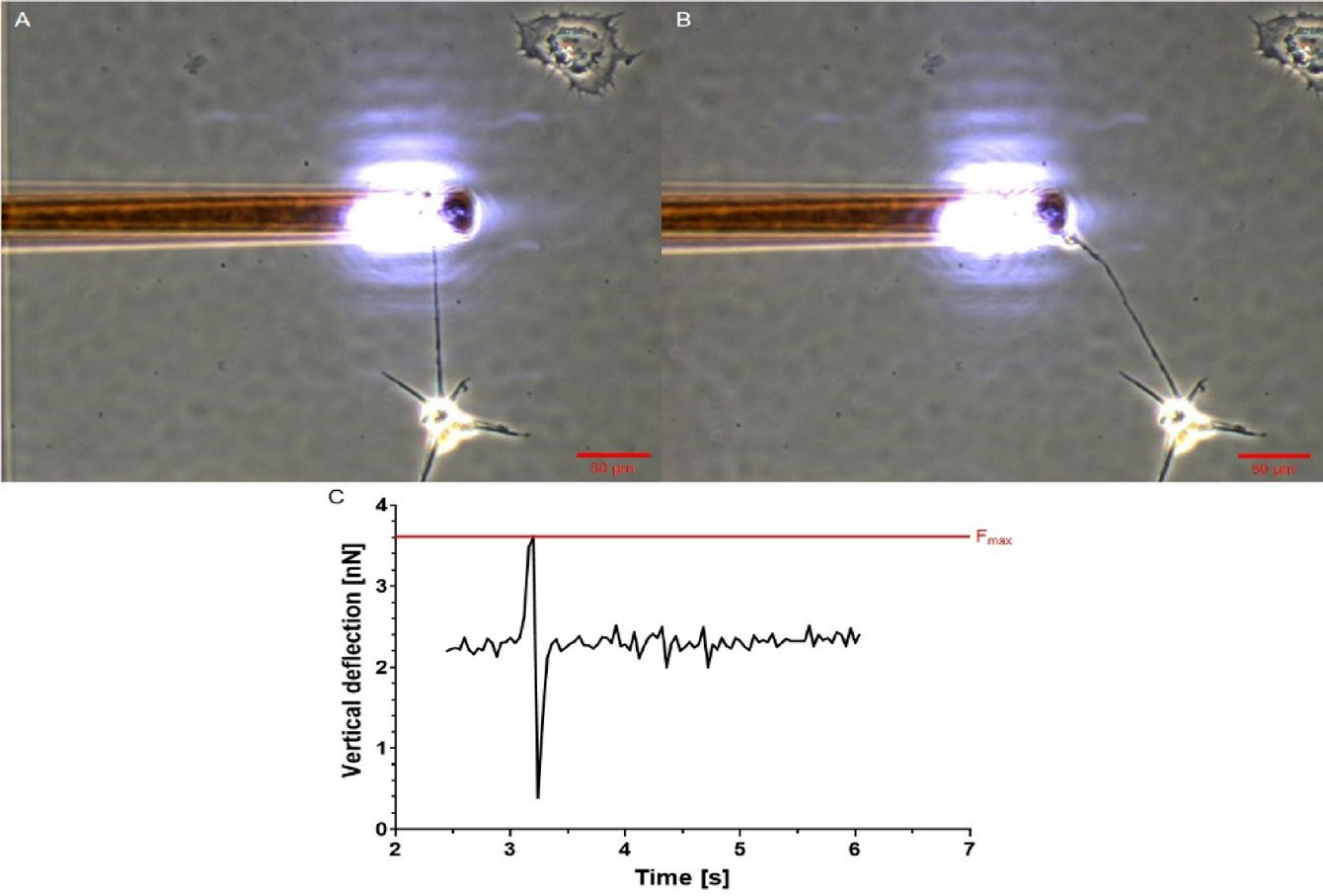
Example of a neurite detachment attempt where hooking and force detection was successful. (**A**) Neurite and cantilever prior to the detachment attempt; (**B**) End of the scan where the cantilever displaced the neurite from the substrate; (**C**) Vertical deflection of the cantilever over the entire detachment process recorded by a real time oscilloscope and the force used for the calculation marked in red.

Consequently, the ideal normal force to detach and successfully track the detachment moment for each neurite varied between 3 and 10 nN. Only neurites with a clear peak in the real time oscilloscope in combination with a visual detachment recorded by the LCD camera were used for the lateral force calculations (Fig. 6A–C).

**Fig. 7:**
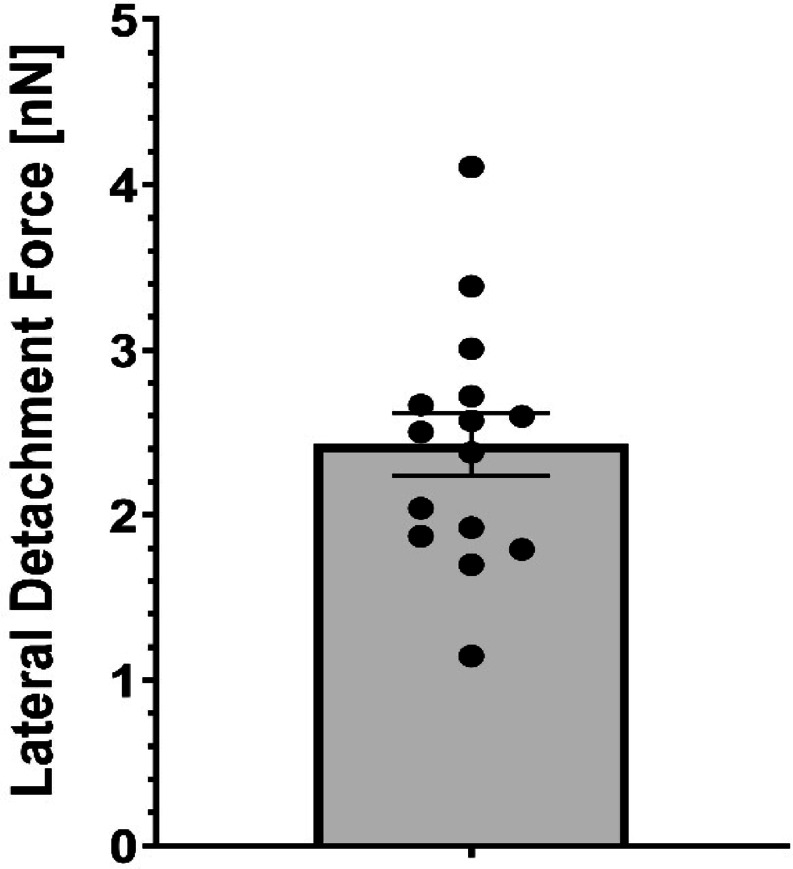
Lateral forces required for the detachment of the neurites from the coverslip substrate calculated with Eq. (1). Each dot represents one neurite. Sample size n = 15.

Forces required to detach the growth cone of a neurite from the substrates surface (Fig. 7) were then calculated with Eq. (1) and the measured maximum vertical deflection values from the real time oscilloscope recordings (Fig. 6C). Mean lateral detachment force from 15 successful attempts (44 in total) was 2.47 ± 0.19 nN.

### Time dependency

**Fig. 8:**
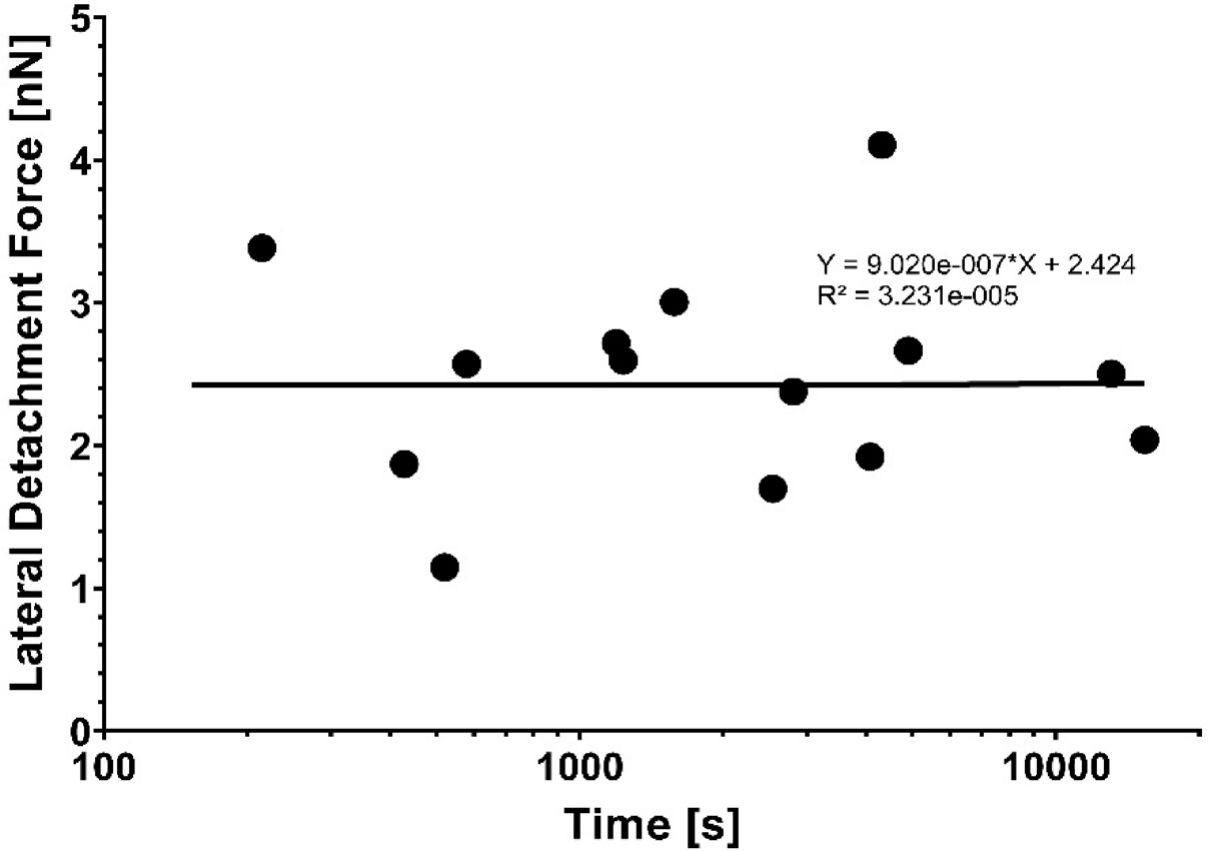
Lateral detachment forces required for detachment over time from the initial overall measurement to the time point of detachment for each individual neurite. Each dot represents one neurite. To improve visualization, a logarithmic scale was chosen for the x-axis. One time point was at t = 0 and therefore not depicted but nonetheless included in the linear regression. Sample size n = 15 (n = 14 depicted).

Under the non-sterile and non-physiological conditions used during AFM measurements, cell viability and adhesion may have been affected. However, the results indicate that the force required to detach a neurite was independent of the time point of detachment. The linear regression showed a slope not significantly different from zero (*p* = 0.984) and an intercept of 2.424 nN, which is close to the mean lateral detachment force of 2.47 ± 0.19 nN (Fig. 8).

### Distance dependency

**Fig. 9:**
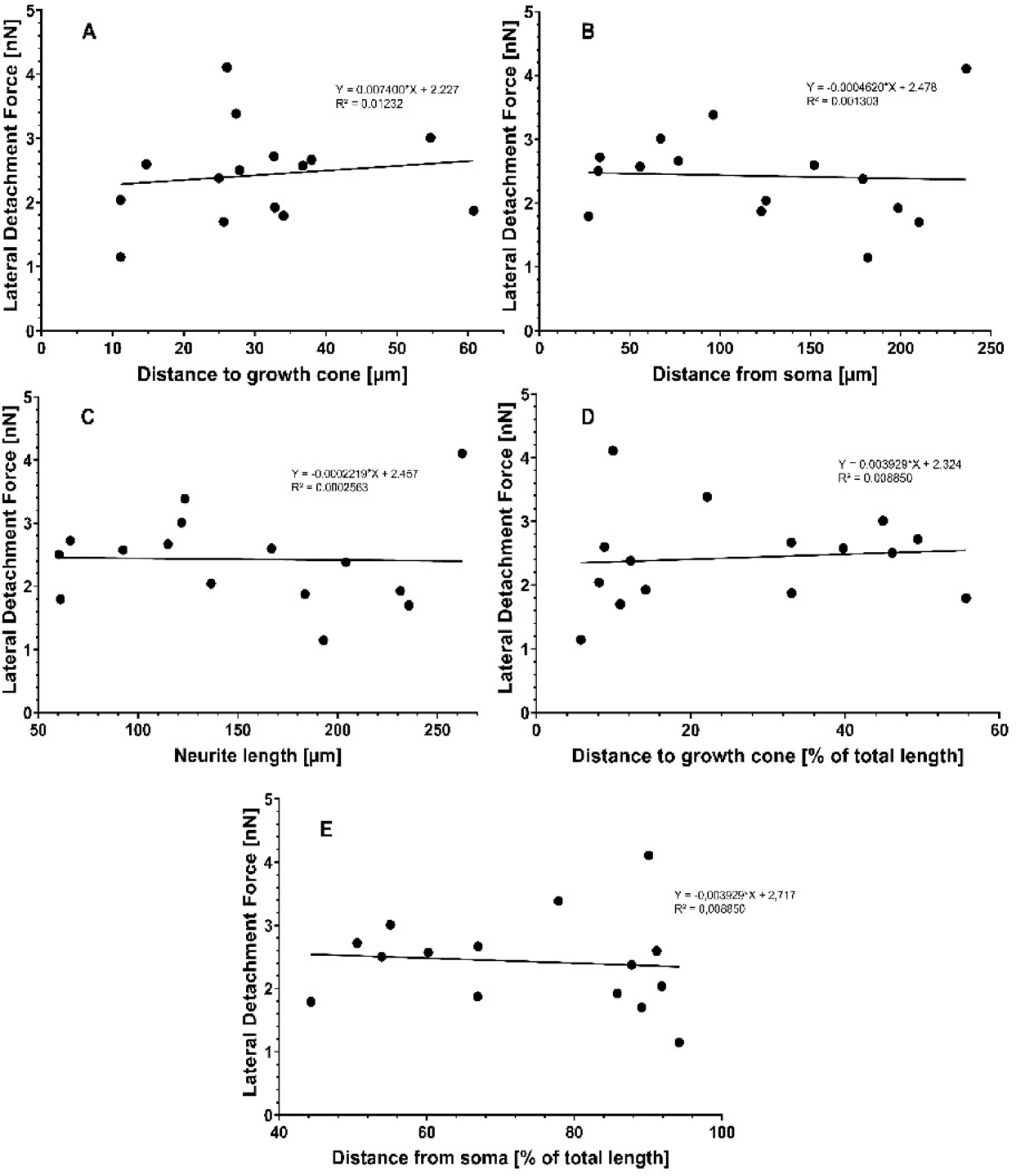
Visualization of the influence on the lateral detachment force of (**A**) the distance between the neurite’s growth cone and the cantilever tip, (**B**) the distance from the neuronal soma to the cantilever tip, (**C**) the total neurite length, (**D**) the percentage from the total neurite length of the distance from the growth cone to the cantilever, and (**E**) the percentage from the total neurite length of the distance from the soma to the cantilever tip. Each data point represents one neurite. Sample size n = 15.

The distance between the cantilever tip and the neurite’s growth cone, both expressed as absolute length and as percentage of the total neurite length, was the first parameter analyzed. (Fig. 9A, D). Although the results suggested a slight increase in lateral detachment force with increasing distance, the slope of the linear regression was not significantly different from zero (*p* = 0.62 (**A**), *p* = 0.76 (**D**)). Therefore, the distance between the cantilever tip and the neurite’s growth cone did not significantly influence the lateral detachment force. In the second distance investigation, the influence of the distance between the neuronal soma of the detached neurite and the cantilever tip, reported in µm and as a percentage of the total neurite length, on the lateral detachment force was evaluated (Fig. 9B, E). The slope of the linear regression was negative, indicating a minimal decrease in force with increasing distance; nonetheless, similar to the previous analysis, the slope was not significantly different from zero (*p* = 0.86 (**B**), *p* = 0.74 (**E**)), and the intercept closely matched the mean of the calculated lateral detachment force (2.478 vs. 2.47 nN). The final length-dependent examination evaluated the relationship between total neurite length and the lateral detachment force (Fig. 9C). The slope of the linear regression was negative again but not significantly different from zero (*p* = 0.93), and the intercept approximated the mean for the lateral detachment force (2.457 vs. 2.47 nN). Therefore, none of the three analyzed distances appeared to influence the force required to detach a neurite.

## Discussion

Purification of SGN from mixed cultures using AraC has previously been employed to determine cellular composition and relative cell-type proportions in primary auditory cell cultures^19^ and is considered essential for long-term in vitro investigation of treatment effects on neuronal survival and outgrowth^20^*.* Use of this mitotic inhibitor enables higher cell densities and extended observation periods without excessive proliferation or detachment of the cellular network due to overgrowth. Despite these advantages, the primary purpose of purification in the present study was to isolate the developed neurites from supporting cells, thereby improving accessibility and facilitating accurate quantification of lateral detachment forces.

Successful neuronal isolation of the culture was achieved, with neuronal survival and neurite outgrowth being consistent with results reported by Schwieger et al.^20^. Purification in combination with neuronal survival peaked at nine days. Prolonged cultivation resulted in widespread cell death (Supplementary Fig. 2). Previous studies have shown that deprivation of neurotrophic factors induce apoptosis in neurons^30,31^. Therefore, the lack of neurotrophic support following the depletion of supporting cells during extended cultivation periods likely caused the loss of neurons and their neurites.

Nine days of cultivation were sufficient to isolate the majority of neurites. However, in larger neuronal clusters, as illustrated in Fig. 3B, supporting cells were still present. Supplementation with neurotrophic factors such as brain-derived neurotrophic factor (BDNF) or neurotrophin-3 (NT-3) may permit extended purification periods^32^. These factors not only promote neuronal survival but also enhance neurite outgrowth, potentially increasing accessibility for AFM measurements. Further purification may also improve neurite identification, as unstained neurites were not always readily distinguishable from glial cell outgrowth. Immunostaining requires fixation of neurons and their neurites, which alters adhesion strength to the substrate and renders lateral detachment force measurements incomparable to those obtained from living cells^33,34^. Although post-measurement staining to confirm neurite displacement was not feasible due to neuronal necrosis caused by the unsterile and non-physiological conditions during AFM measurements, the adhesion strength of the neurites was not significantly affected by these conditions (Fig. 8). Future measurements under physiological conditions (e.g. use of cell culture medium, controlled temperature at 37 °C, regulated CO_2_ concentrations) could enable immunostaining following the measurements.

Clifford and Seah have stated that the nominal spring constant and resonance frequency provided by the manufacturer can vary significantly between cantilevers. Therefore, calibration of the cantilever is essential to minimize systematic errors in the force recordings^35^. Furthermore, Pimenta-Lopes et al. demonstrated that external factors, such as temperature fluctuations or subtle structural defects, can influence the spring constant and thus affect the force calculations^36^. In the present study, although the same cantilever was used for every measurement, it was calibrated individually prior to each session to account for day-dependent variations and to detect potential mechanical defects that could alter the cantilever sensitivity and spring constant. Additionally, the cleaning procedure of the cantilever after each measurement session helped to preserve its condition.

Measurements of detachment forces of cellular structures from non-biological materials are widely conducted^21,22,24,28^. These studies primarily focus on the influence of different topographies on adhesion strengths and the forces required to displace an entire cell. In the present study, we instead aimed to establish a reliable method for detaching neurites from substrates. Because neurites are significantly smaller in size than cell types such as osteoblasts or chondrocytes examined in previous studies and are normally ensheathed by supporting cells, the forces required for lateral detachment were comparatively low (2.47 ± 0.19 nN), in contrast to reported values of 43 ± 21 nN for osteoblast filopodia on titanium and 74–185 nN for chondrocytes, depending on seeding time^21,28^. Consequently, a cantilever with the elevated sensitivity was required for accurate force detection. Despite the difference in size compared to whole chondrocytes or osteoblast filopodia, the measurement method and the calculation of the lateral detachment force have also been applied to smaller biological structures such as *Escherichia coli*, which have a diameter ranging from 0.7 to 1 µm depending on external conditions^24,37^. With reported detachment forces of 0.764 ± 0.167 nN on polished stainless steel and 0.639 ± 0.136 nN on glass substrates coated with poly-l-lysine^24^, size and detachment force were more comparable to those of neurites (0.5 to 1.5 µm in diameter^38,39^ and 2.47 ± 0.19 nN). Although the equation is applicable to different cells and structures, direct comparisons between them remain challenging. Morphological differences and different adhesion mechanisms introduce multiple confounding factors that influence the adhesion strength and, consequently, the measured detachment forces. Here we present a methodological approach to determine the forces required for the detachment of neurites derived from SGNs. Further investigations are required to evaluate the influence of different surface properties and the adhesion behavior on the detachment force of neurites.

Neurite detachment was performed using the contact manipulation mode of the AFM system, in contrast to previous studies that relied on the contact scanning mode^22,24^. In scanning-based approaches, scanning of an area was used first to locate cells and subsequently to detach them through repeated scans until displacement from the surface occurred. For neurite ends, however, this strategy is not suitable, as the low forces applied during localization alone may be sufficient to induce detachment. Moreover, the scanning mode relies on sequential linear scans over a defined area to acquire topographical information. Such an approach is problematic for neurite measurements because neuronal outgrowth is a complex biological process involving bridging between distinct bioactive sites^40^ and does not implicate consistent connection to the substrate throughout the entire length^41,42^. In mixed primary neuronal cultures, neurite extension is strongly influenced by direct contact with supporting cells, and only discrete growth cone regions engage with substrates, whereas the neurite shaft often lacks continuous adhesion. Consequently, most of the neurites form only a limited amount of attachment points to the substrate and predominantly connect to other cells. Following depletion of supporting cells by the mitotic inhibitor, neurites therefore remain hanging loosely between their lasting connection points. Accordingly, in this study, the objective was the targeted detachment of individual neurite ends. Therefore, the precise positioning of the cantilever tip near the neurite’s growth cone was essential. Other studies analyzed repetitive detachment of neurites from poly-l-lysine coated glass substrates and reported initial normal forces required for detachment of 4.15 nN, which is in the range of the normal forces used in the current study^43^.

Although positioning of the cantilever was important to achieve successful detachment, the results exhibited that neither the distance between cantilever tip and the growth cone of the neurite or the neuronal soma, nor the total neurite length affected the forces required for detachment (Fig. 9). This may be attributed to the limited number of remaining substrate connection points along the neurite, leaving the majority of adhesion strength concentrated at the growth cone end, which was adherent to the surface.

The manipulation mode of the AFM allows scanning along a user-defined path of interest within a 100 × 100 µm measurement area. In combination with the exact cantilever positioning, this approach enabled detachment of neurites in a single scan, similar to the method described by Nguyen et al.^21^. Applied force, scanning speed and cantilever adjustment frequency to the surface topography can be predefined within the software. Thus, the use of contact manipulation mode provided enhanced control over force application and improved reproducibility of the detachment measurements.

Effective neurite detachment required a combination of high scanning velocity and constant applied forces to enable hooking of the neurite and rapid separation of its end from the substrate. Vertical forces in the range of 3–10 nN yielded the most reliable results, producing clear force peaks at the moment of detachment. At lower forces, the neurites hooking failed and the cantilever merely scanned over it, whereas at higher forces no distinct detachment peak was detectable (Figs. 4, 5). The upper limit for detectable peaks was set to 10 nN, and the stepwise increase in normal force starting at 3 nN ensured that each neurite was detached using the minimal force required. Furthermore, to improve accuracy of the calculated data, only neurites exhibiting complete and detectable detachment were included in the analysis. Additionally, new neurites were selected when complete hooking was unsuccessful, but resulted in a visible displacement, or when the covered distance was insufficient to achieve full detachment. Although, these stringent selection criteria improved the accuracy of the analyzed data, they also increased the proportion of unsuccessful detachment attempts to the successful ones. One potential explanation for the variation in normal force required for detachment is the attachment of neurites at distinct bioactive sites. Hooking may require less force when applied between two attachment points, where the neurite is more loosely suspended, compared to regions near the growth cone, where it is attached to the substrate. Nevertheless, despite differences in the normal force required for hooking, the results indicate that the lateral detachment force was independent of the hooking position.

While prior studies used scanning speeds of up to 40–50 µm/s^22,28^, best detachment results were achieved with 20 µm/s in the current study. At faster scanning speeds, the cantilever tip did not remain in contact long enough to enable hooking of the neurite, whereas at slower velocities the flexibility of the neurite allowed it to adapt to the deformation caused by the cantilever tip thereby preventing effective hooking and detachment.

Although the manipulation mode allowed the detachment in a single scan, the maximum distance that could be covered was limited to 100 µm. Neuronal outgrowth over several days can form networks connecting neurons and other cells, extending neurites over several hundred micrometers to facilitate information transmission. Mechanically disrupting the connection between two cells could provide valuable insights into the adhesion strength of neurites between connected cells on the implant surface, rather than directly to the substrate. However, due to the distance limitation of a single scan, we were only able to investigate the lateral forces required to detach the end of individual neurites, without examining more complex outgrowth. AFM systems equipped with a motorized sample holder may permit further scan distances, enabling measurements of the disruption forces for neurites connecting two neurons.

Bello et al. demonstrated that the topography of titanium surfaces affects lateral detachment force, and thus adhesion strength^28^. Here, we focused on purification of the SGN mixed culture and therefore used coated coverslips, which are commonly employed in in vitro auditory treatment investigations^6,10,20,44^, although they do not replicate the actual surface of CIs. Using materials present in electrode arrays, such as platinum for the contacts or silicone for the electrode array, may influence lateral detachment forces. As reported by Reich et al. fibroblast growth is reduced on silicone compared to platinum^45^. If this effect also applies to neurons and neurites, their adhesion strength would be decreased on silicone compared to platinum, resulting in a reduced lateral detachment force. Furthermore, the coated coverslips used in the current study provide an optimized surface for neuronal attachment and outgrowth. Consequently, lower lateral detachment forces would be expected on platinum surfaces; however, these surfaces have yet to be evaluated.

## Conclusion

In the current study, a method for measuring neurite adhesion strength was established. Using a purification protocol for primary SGNs, neurites were isolated from supporting cells through cultivation with the mitotic inhibitor AraC over nine days. AFM measurements were successfully performed to determine the lateral detachment forces required to separate the adherent, isolated neurite’s ends from precoated glass substrates. Measured forces were low compared to whole cells or other cell types and independent of exposure time to non-physiological conditions, the position of detachment and the total length of the neurite. These results provide a foundation for further investigations into the effects of neurite hooking location and substrate surface properties on adhesion. For further investigations, physiological conditions during measurements are mandatory to ensure maintenance of cellular viability throughout the measurement window.

## Supplementary Information

Below is the link to the electronic supplementary material.

Supplementary Material 1

## Data Availability

The datasets generated during and/or analysed during the current study are available in the figshare.com repository, 10.6084/m9.figshare.31746757.
