# Supplementary material for: Method for AFM investigation of lateral forces required for the surface detachment of neurites
Source: Sci Rep. 2026 Jul 7;16:20999. doi: 10.1038/s41598-026-60250-1 (PMC13342227; doi:10.1038/s41598-026-60250-1)
Supplement: Supplementary file 1 — Supplementary Material 1 [file 41598_2026_60250_MOESM1_ESM.pdf]

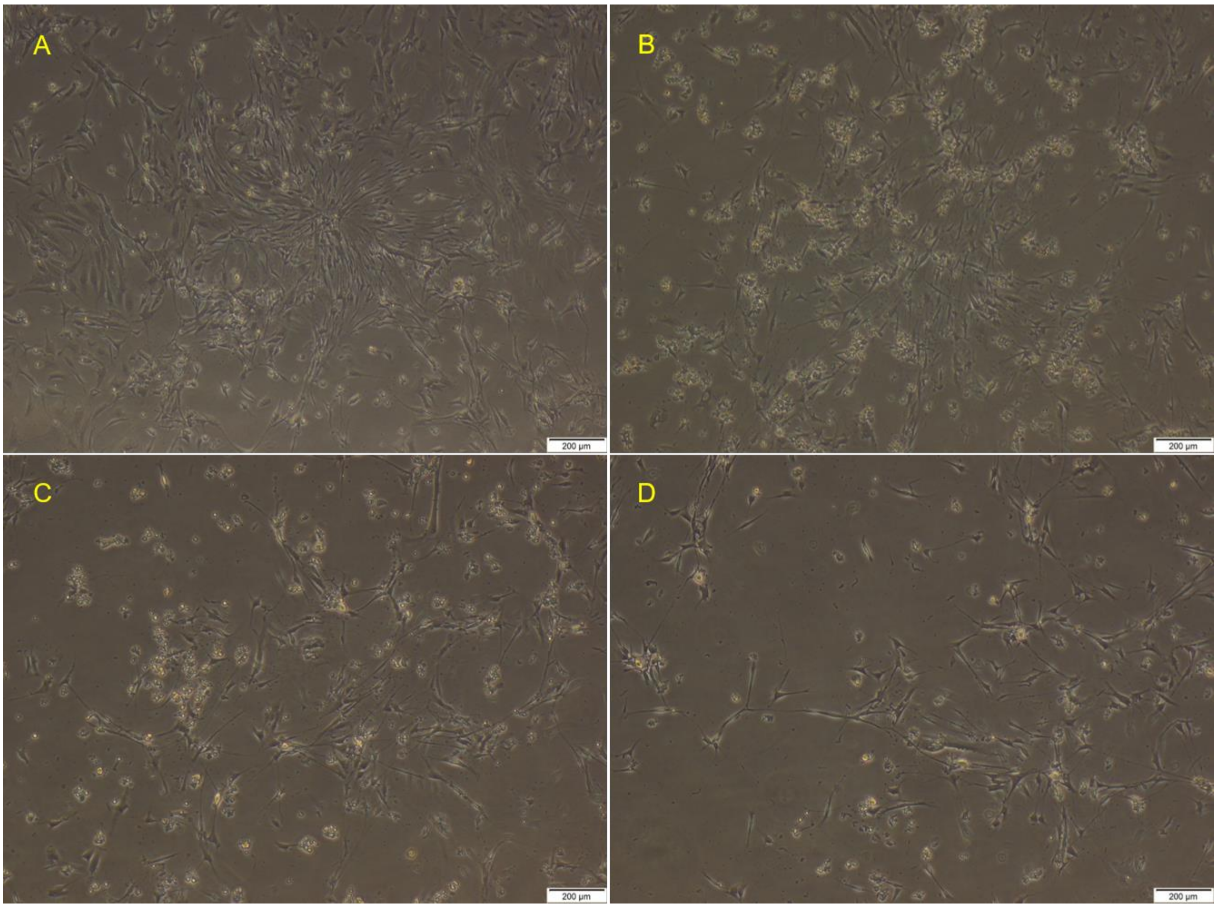

Supplementary Figure 1 SGN purification progress over the course of 8 days. Images illustrate the progress of the same well before cell culture medium change after (**A**) 2, (**B**) 4, (**C**) 6 and (**D**) 8 days. Scale bar indicates 200 µm.

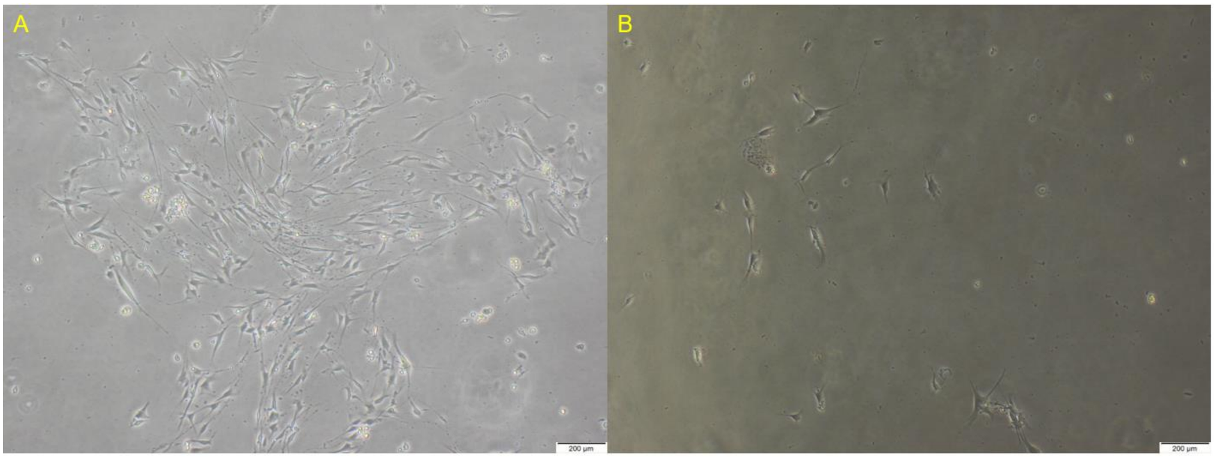

Supplementary Figure 2 Images of a representative well where the cultivation period with AraC was extended to 14 days. Images were taken before cell culture medium exchange at (**A**) 5 days and (**B**) 14 days of cultivation.
